# Precision Public Health and Structural Racism in the United States: Promoting Health Equity in the COVID-19 Pandemic Response

**DOI:** 10.2196/33277

**Published:** 2022-03-04

**Authors:** Lester Darryl Geneviève, Andrea Martani, Tenzin Wangmo, Bernice Simone Elger

**Affiliations:** 1 Institute for Biomedical Ethics University of Basel Basel Switzerland; 2 University Center of Legal Medicine University of Geneva Geneva Switzerland

**Keywords:** precision public health, structural racism, COVID-19, pandemic, social justice, health equity, SARS-CoV-2, stigma, discrimination, disparity, inequality, precision health, public health, racism, inequality, equity, mortality, morbidity

## Abstract

The COVID-19 pandemic has revealed deeply entrenched structural inequalities that resulted in an excess of mortality and morbidity in certain racial and ethnic groups in the United States. Therefore, this paper examines from the US perspective how structural racism and defective data collection on racial and ethnic minorities can negatively influence the development of precision public health (PPH) approaches to tackle the ongoing COVID-19 pandemic. Importantly, the effects of structural and data racism on the development of fair and inclusive data-driven components of PPH interventions are discussed, such as with the use of machine learning algorithms to predict public health risks. The objective of this viewpoint is thus to inform public health policymaking with regard to the development of ethically sound PPH interventions against COVID-19. Particular attention is given to components of structural racism (eg, hospital segregation, implicit and organizational bias, digital divide, and sociopolitical influences) that are likely to hinder such approaches from achieving their social justice and health equity goals.

## Structural Racism and Defective Data: Health Equity Threats for the Public Health Response Against COVID-19

Structural racism refers to “[a] system in which public policies, institutional practices, cultural representations, and other norms work in various, often reinforcing ways to perpetuate racial group inequity” [1]. Structural racism affects all social determinants of health. Its impact was accentuated and made more visible with the COVID-19 pandemic. Indeed, the US Centers for Disease Control and Prevention recognized the centrality of the social determinants of health in the disproportionate impact of the pandemic on racial and ethnic minority groups. In addition, they acknowledged the pervasive contributing influences of (structural) racism across each one of these determinants [2]. Undeniably, since the start of the pandemic, a cornucopia of examples suggesting the impact of structural racism has surfaced [3]. This was particularly evident in countries from which statistics are presently available, such as the United States, showing devastating consequences for the health of racial and ethnic minorities [4]. For instance, as of November 2021 in the United States, the risk of dying from COVID-19 for non-White groups (except for the Asian non-Hispanic community) was about twice that of White people [5]. Such inequities can be partly explained by structural vulnerabilities influencing access to and quality of care offered to these racial/ethnic minority groups.

The structural vulnerabilities of some racial and ethnic minority groups to COVID-19 stem from political and social influences, which were observed, for instance, to have a greater impact on their health than individual choices made [6]. These influences led to the normalization of discrimination, stereotyping, and prejudices, and ultimately impacted racial minorities’ health and access to quality care [7]. Indeed, studies have demonstrated the existence of implicit racial/ethnic biases among health care professionals, and their presumed impact on the quality of care due to suboptimal patient-provider interactions with racial/ethnic minorities (see review [8]). For instance, emerging evidence based on billing data started indicating disparities in COVID-19 testing among racial and ethnic groups, with African Americans being less likely to be offered COVID-19 testing than Whites, even when presenting with similar symptoms such as fever and cough [9]. Aside from individual health care professionals, health care institutions and organizations can also harbor implicit biases regarding their approach toward minority groups. It was highlighted that testing centers for COVID-19 in the United States were predominantly located in wealthy and White neighborhoods, which further limits access to health care for people located in poor neighborhoods [10]. These implicit provider and institutional/organizational biases could be an additional source of structural health disparities for ethnic and racial groups during the COVID-19 pandemic [11]. Indeed, average threshold metrics for public health interventions (eg, those used as indicators for closing or opening schools/businesses) are biased if COVID-19 testing is carried out mostly in wealthy neighborhoods—where viral transmission rates are actually lower—and deprived neighborhoods (where residents are predominantly minorities) are insufficiently tested [12].

Additionally, public health recommendations for containing the spread of COVID-19 have been particularly difficult for racial and ethnic minority groups to implement, partly due to the downstream influences of structural racism [3,13]. As rightly noted by Krieger [14], racial and ethnic minority groups constitute a good percentage of low-wage workers, making them more vulnerable to the effects of the pandemic. They often live in crowded multigenerational houses (often a consequence of racial residential segregation and redlining policies—two historical examples of structural racism [15,16]), with neither the possibility of working remotely from the safety of their homes nor adequate access to COVID-19 testing and treatment (eg, because minorities are less likely to be insured) [3,17].

Other indicators of how structural elements determine health inequity for racial/ethnic minorities during the COVID-19 pandemic have been highlighted by some seroprevalence studies. For instance, a large-scale nationwide study carried out in the United States showed higher seropositivity rates (2- to 3-fold higher) for SARS-CoV-2 antibodies in dialysis patients residing in Hispanic and non-Hispanic Black neighborhoods in comparison to those residing in White (non-Hispanic) neighborhoods, and a 2-fold higher seropositivity for those living in poorer neighborhoods [18]. This study and others (eg, [19]) challenge the underlying assumption made elsewhere that “the risk of infection is homogeneous within the population” [19]. Such implicit assumptions are not only detrimental to the efforts made to contain the virus but they may also be a manifestation of structural racism itself. Indeed, one of the defining characteristics of structural racism is its *invisibility* to the dominant racial/ethnic group [20]. The situation also highlights the need for more comprehensive data on the impact of the COVID-19 pandemic on racial and ethnic minorities, in particular for those living in disadvantaged neighborhoods.

In that regard, eminent public health researchers have raised the alarm regarding missing or incomplete data on racial/ethnic minorities [14,21]. Indeed, data on the impact of COVID-19 on racial and ethnic minorities were not systemically and uniformly collected across the United States [22]. This resulted in the skewing of our understanding of the deadly evolution of the virus within these communities, while hampering the ability to provide them with timely and adapted public health interventions and care [14,23]. Although US state and local public health departments were required—at latest by August 1, 2020—to report demographic data for COVID-19 cases, Krieger and colleagues [23] have shown that, despite the new reporting requirements, compliance was far from being achieved and much work still needs to be done in this regard.

In the COVID-19 era, the underreporting and inadequate reporting of racial and ethnic information create data gaps that hamper the proper functioning of public health institutions in initiating culturally appropriate measures to prevent the spread of the virus in these communities [24]. Given the fast pace at which the pandemic is evolving and the continuous need for updated public health responses, a paramount question is how these data gaps and structural racism could influence public health practice, in particular when innovative data-driven approaches are being considered as complementary to traditional public health interventions. In this paper, we thus reflect on how structural racism in the health care/public health domain and the defective collection of data on racial and ethnic minorities could undermine the health equity and social justice goals of precision public health (PPH) interventions.

## Structural Racism and Defective Data: Potential Impact on PPH

To respond to this worrying public health situation while making use of existing and emerging data sources, Rasmussen and colleagues [25] argue for the need to implement PPH interventions as an additional tool to fight the pandemic. PPH means “the application and combination of new and existing technologies […] to tailor preventive interventions for at-risk groups and improve the overall health of the population” [26]. Horton [27] is even more explicit in highlighting the importance of data in PPH and characterizes it as being “about using the best available data to target more effectively and efficiently interventions of all kinds to those most in need.” At least two distinct approaches to PPH exist. The first one is a reductionist version, where PPH focuses solely on the use of genetic information to tailor interventions to specific subgroups of the population (with the risk of neglecting foundational considerations of public health such as the impact of the social determinants of health). The second (wider and more encompassing) version does not limit itself to genetic information, but also considers other sources of data (eg, big data, granular population surveillance data, and data from mobile apps) to guide public health practice [25,28]. Given the limits of focusing solely on genetic data to guide interventions (eg, risk of exacerbating existing health inequalities [29]), we find the second version of PPH more promising, as it uses a plethora of data sources [28], in particular if it will ally both high-risk strategies and population-based approaches to maximize the impact of public health interventions [30].

Although still in its infancy [31], PPH has already shown promise in the fight against COVID-19. For instance, at the start of the pandemic, pathogen genomics such as whole genome sequencing analysis, coupled with epidemiological data, was successfully used in the Netherlands to not only monitor the emergence of local or regional clusters of SARS-CoV-2, but also to help in understanding transmission patterns while guiding public health interventions in breaking the chain of transmission of SARS-CoV-2 [32]. Additionally, some innovative sources of digital data are also being used to reduce the transmission of SARS-CoV-2. The usefulness of participatory disease surveillance systems has already been demonstrated for the early detection of other transmissible diseases such as influenza-like illness [33]. In such systems, citizens can actively get involved in the public health response by directly reporting COVID-19–related symptoms via mobile apps or digital platforms [34]. Furthermore, the use of COVID-19 contact trackers, whereby cellphone tracking data are used to alert people who might have been in contact with an active case of COVID-19 [35], is another example of how PPH can be helpful in a pandemic context.

Given the disproportionate burden of the pandemic on racial/ethnic minorities, as previously argued, it would then be legitimate to consider them as part of those “most in need” [27] and thus principal supposed beneficiaries of PPH interventions during the COVID-19 pandemic. However, given the aforementioned issues related to structural racism and the skewed collection of data on minorities, it is important to consider the following two issues before the widespread deployment of PPH interventions in connection to COVID-19. First, one must reflect on how structural racism can influence the generation and use of data sets in public health practice (eg, data racism) [36]. Second, it is critical to explore how the use of certain data-driven technologies—which are becoming essential components of PPH—could lead to novel racial/ethnic discrimination in public health interventions for those “most in need” [27].

One set of technologies that has the capacity to both improve or worsen health inequities between ethnic and racial groups if employed in clinical care and PPH interventions is machine learning [37]. Machine learning can be defined as “a branch of artificial intelligence (AI) focused on building applications that learn from data and improve their accuracy over time without being programmed to do so” [38]. There are 3 main classifications of machine learning approaches, namely supervised, semisupervised, and unsupervised learning, depending on whether the machine learning algorithms are trained on labeled, semilabeled, or unlabeled data sets, respectively [39]. If such technologies are used in PPH, one of the important aspects to consider is the appropriateness of data sets used to train machine learning algorithms, in particular if they are to be used in a population that is either underrepresented in the training data sets or has long been systemically disadvantaged and marginalized [40,41]. Indeed, individual and societal biases can be encoded in big data and other training data sets destined for public health practice and medical care [37,40]. This phenomenon is sometimes described as data racism, a term that refers to “the multiple systems and technologies - deployed in a range of fields - that either primarily target or disproportionately impact migrants and people of [color]” [36]. To better explain how data racism—combined with structural racism—could impact PPH interventions through machine learning techniques in the future, one can look at the unfortunate experiences emerging in the field of data-driven predictive policing [42].

PPH and predictive policing can be compared since they function according to analogous principles. Indeed, one of the foreseeable goals of PPH is to forecast disease outbreaks and identify hotspots or subgroups of the population for tailored interventions based on big data predictive analytics [43]. Similarly, predictive policing aims at forecasting the likelihood of a crime being committed in a specific location, to then prioritize focused police interventions in certain at-risk areas (eg, by having more frequent police patrols) or on people having some prespecified characteristics deemed relevant by the used software. Existing predictive policing software companies, such as PredPol, aspire to also be fair, since the “starting point is data: objective, agreed-upon facts that can be used to guide the discussion” [44,45]. PredPol claimed to provide fairer and more objective risk evaluation and predictions regarding crimes than subjective police assessment [44]. However, Richardson and colleagues [42] argue that, although PredPol took significant actions to reduce bias in their data sets when training their machine learning algorithms (eg, by excluding traffic citation data and data on drug-related crimes), such measures still do not capture the whole complexity and diversity of police interactions where bias can be introduced into the data. They also highlighted the methodological difficulties for vendors of such technologies to identify “these problematic practices and policies in real-time; therefore, any system that includes recent or live data may be subject to additional undocumented biases” [42]. Consequently, the alleged promise of being fair cannot always be kept, since data sets on which predictive policing is based often present relevant fallacies.

We can reasonably expect that PPH interventions might encounter an analogous set of problems, in particular considering the previously discussed COVID-19–related data crisis for racial and ethnic minority groups. Indeed, within the precincts of structural/data racism, machine learning algorithms will likely replicate some degree of discrimination unless appropriate measures are taken to address the situation [40]. Therefore, it is important for machine learning developers to have an adequate understanding of structural racism and its potential real-world ramifications through their software [46]. This could help ensure that the developed machine learning algorithms both advance public health utility and promote a fair distribution of resources along racial and ethnic lines, while minimizing the risks of worsening health inequities [46]. However, it is also important to note that addressing the technical flaws of machine learning algorithms is catering only to the downstream consequences of structural racism and therefore this cannot be the silver bullet to reduce health inequities between racial and ethnic groups. To bridge the disparities between racial and ethnic groups will likely require changes at the societal, institutional, and individual levels, so that the upstream influences of structural racism are mitigated [47]. A few additional potential solutions that can help in tackling algorithmic biases have been discussed in a previous publication [40].

Aside from technological considerations, it is also paramount to tackle the low representation of racial/ethnic minorities (in particular those of African descent) in AI and machine learning communities. This can also be considered a consequence of structural racism in industrial and academic settings [48]. Racial and ethnic diversity in these communities could help safeguard against blatant and implicit discrimination toward minorities, even if these minority researchers could themselves be subsequently exposed to consequences from the power structures in place. Indeed, it is documented that some of the effects of structural racism in the workplace also involve microaggressions toward these minorities (eg, harassment, disrespect, racial slurs) by their White colleagues, which undermine their capacity to work, while operationalizing the corporate culture of maintaining racial hierarchy [20,49].

There are also other means by which structural racism could impact the generation of data for PPH during the COVID-19 pandemic. One of them is through the racial and ethnic digital divide, whereby minorities, as a consequence of their often-lower socioeconomic status, would be less likely to engage in PPH activities due to poorer internet access [50]. According to the Pew Research Center, in 2019, White individuals still have better access to the internet in comparison to other racial/ethnic groups (92% versus 86% in Hispanic individuals and 85% in African Americans) [51]. Therefore, public health surveillance systems relying on internet-collected data (eg, social media mining to guide interventions against COVID-19) may be particularly vulnerable to the underrepresentation of minority groups in the gathered data sets. The problem might also be that of overrepresentation of minorities in internet-collected data. For example, a recent study has found that—despite worse internet access—ethnic/racial minority groups are more likely to post COVID-19–related information on social media, possibly due to a “reversal of digital divides” or the fact that the pandemic has disproportionately impacted racial and ethnic minority groups, and that social media is often perceived as a coping strategy for stress and for obtaining community support [50]. Either way, the issue remains that proper public health surveillance should ensure the ethnic and racial representativeness of the collected data before initiating any public health intervention [50], or at least perform some sort of data adjustment to account for the selection bias.

Lastly, it cannot be ignored that structural racism could lead to more biased data sets for ethnic and racial minority groups because of the influence of sociopolitical factors on the functioning of public health institutions [52]. It is important to note that institutional (or structural) racism can influence the functioning of institutions in a number of ways (ie, at individual and organizational levels). For instance, institutions can either become increasingly racialized and adopt racist motivations (eg, excluding or limiting employment of qualified minorities in decision-making positions, or employing a few of them only, as part of racial capitalism [53], to showcase that they are dedicated to diversity) or they can be burdened by other organizational structures that impede their achievements in terms of racial and health equity (eg, lack of resources and low care given to people of color) [52]. Indeed, it is well known that hospitals in marginalized neighborhoods (eg, those with a high percentage of people of color) are underfunded, understaffed, and sometimes filled with less skilled health care professionals, which then limit their ability to offer services and treatment comparable to those offered in other neighborhoods [15,54].

Therefore, such public health institutions could be hampered in generating high quality data on racial and ethnic minority groups due to (1) the limited resources dedicated to these groups, (2) the unrepresentative racial and ethnic composition of their decision-making teams and health care professionals, leading to viewing racial disparities from a “White framing,” (3) the lack of adequately trained professionals to fulfill these duties, and (4) hospital segregation—that is, the refusal of some of the most resourceful hospitals to treat racial/ethnic minority groups suffering from COVID-19 due to their lower socioeconomic conditions (eg, inferior health insurance plans, which is also a known consequence of structural racism) [40,54,55]. The unfortunate consequence could be that of contributing to the generation of biased data sets that do not reflect the lived reality of racial and ethnic minorities in facing COVID-19. Therefore, PPH interventions relying on these data sets would likely be limited in their effectiveness and health equity could never be achieved for all racial and ethnic groups, unless these different parameters are given due consideration.

## Conclusions

In this article, we have highlighted the role of structural racism and the presence of defective data for racial and ethnic minorities as important factors to consider in designing ethically acceptable public health policies during and after the COVID-19 pandemic. Moreover, we have discussed PPH, an innovative approach to tackle public health issues such as COVID-19, which can however encounter several ethical challenges if the aforementioned issues of structural racism and defective data collection are not tackled. Our aim is not that of discouraging the use of PPH, which we consider as an important new element in the public health toolbox that deserves to be fully implemented to fight the COVID-19 pandemic; rather, we want to highlight a few issues that need to be considered by policy makers and scientists developing PPH measures to make sure that their efforts to improve public health do not ignore the danger posed by structural racism and defective data on minorities. In this regard, we join our voices to those cited in this article on the importance of having improved, harmonized, and nationwide data collection systems that are as free as possible from the influences of structural racism and inclusive of all racial and ethnic groups. Although insufficient on their own to promote health equity among racial and ethnic groups, these measures could be an important contribution to the effective and nondiscriminatory use of PPH approaches that are inclusive of the racial and ethnic composition of the societies in which they are deployed. PPH approaches deserve to be better planned and their effectiveness critically assessed, and this will not be achieved unless their data and technological foundations are deep-rooted in health equity and social justice.

## Abbreviations

AI: artificial intelligence
PPH: precision public health
